# The decay in Indian education and society: Are we responsible for it?

**DOI:** 10.4103/0973-6247.59383

**Published:** 2010-01

**Authors:** Nabajyoti Choudhury

**Affiliations:** *Asian Journal of Transfusion Science, India*

Let us discuss something which is not related to any field of medical sciences, not by any means near to transfusion medicine. We will discuss the word “mafia” and how this word exerts undue influence in our lives. I want to clarify in the beginning that this discussion is not related to social/ political/ under-world mafia existing around us. What we are going to discuss is about a group of people influencing our working whether at work or in our professional life.

What is the meaning of “mafia”? As mentioned in the Oxford Dictionary, it is “a powerful group who secretly influence matters.” Do you feel sometimes suffocated or helpless because of the actions of some other persons where you cannot do anything? Whenever, anybody influences decision-making, that person may be a mafia, literally. We will discuss about these people against whom we are helpless or cannot do anything! The discussion will be on helplessness in different sections like medical education, medical services, government agencies/regulators, social organizations, and another new dimension like corporate bullies.

You may remember few instances in your academic/education life where you were pathetically helpless. It may be due to system failure because of wrong system in place or because a group of people influence decision from outside. In our education system, there are many dubious academic institutes across the country. Most of them are affiliated to state or central government universities. Governments allow this type of technical educational institute to promote educations and increase pool of skilled manpower as our growing population. The intention is good to promote higher education at an affordable price with less red tapism. But the system has been exploited by many institutes in order to make money. These self-financed colleges take obnoxious sums of money to get admission in different courses. There are no rates or rules fixed to get admission. It is the amount of money parents can shell out. Most interestingly, you will not get any money receipt in these types of transactions. Public is really helpless in front of these people when they need higher education for their children. One of my friends explained his experience when he approached one of the little known dental colleges to secure admission for his daughter. The Principal informed the admission rates are INR 30,00,000 as a “donation” and there was no system of written money receipt. When my friend asked what if somebody refused to acknowledge the payment in future, the authority replied that everything was on “trust.” What do you call these people? I do not find any better word than “medical mafia.”

What to talk about one administrator from one dental college? If we look around, even regulatory agencies are no better. Our government in the era of globalization is now talking of bringing an end of “license-raj.” It is true that whenever there are some involvement of inspectors, subjectivity and corruption becomes a synonym. This is observed among first rank inspectors to the higher level officials. If you have experienced in obtaining blood bank license, you will know how they influence medical decisions. Many a times, inspectors force blood bank personnel to bow-down to a humiliating limit as per their whims and fancy. It also depends on what type of blood banks you are managing! If you are from government sector, you are lucky. But if you are from NGO or private sector, the attitude towards assessee is “you are a thief unless proved otherwise.” This is an endless story with blood bank regulatory agencies across the country. However, of late the situation has improved slightly due to the tough action in higher level and also for proactive attitude taken by higher-ups.

The saddest part is that the same type of corruption termite has started eating up our medical educational system. You may have remembered about comments passed by the Union Health Minister about serious corruption problems in Medical Council of the country. The unnecessary strictness (harassment?) faced by individual medical colleges, especially in private sector, is very sad. If you approach to the right person and if you know how to manipulate, it is easy to get recognition of your medical college. It is even interesting to note how state governments manipulate. Just before any MCI inspections, there is temporary transfer of huge number of teaching faculty from one government medical college to another. Once MCI inspection is over, same faculty is transferred back after few months. MCI inspectors also know what is happening and turn a blind eye being a government institute. However, in the same situation at private medical colleges, corruption prevails. It is worth mentioning that the President of the Council was implicated by the court on serious charges who came back only after a long legal battle. Under this circumstances, who do you think influences decision from outside; the Medical Council, the head of the Council, or various state governments?

Other sphere of medical field where we observe bullying tactics or presence of mafia is in scientific societies. Ironically, all members are highly educated and technically competent professionals. It is natural to observe differences among members of scientific societies. However, strong differences, which lead to obstruction of progress, are suicidal. All such societies are registered under the Registrar of Societies. These are democratic institutions and they are supposed to follow certain democratic norms as per individual constitutions. However, in many instances, members try to bypass the system. Office bearers are elected by democratic process. Sometimes elections are not hold or sometimes influential people try to bypass election process. Unfortunately, active politics has entered in many scientific organizations. Many a times members are divided as per political affiliation and regional factors also play strong role. It brings serious dissatisfaction and ultimately revolt in the organization. We must learn to respect our colleague and promote the cause of the organization rather than using organizations for other interests.

A recent incidence of presence of “educational mafia” in India has been proved beyond doubt when the Union government derecognized 44 (53.7%) deemed universities in the country. Out of 82 deemed universities in the country, only 38 were found to be eligible to retain the status. Few important issues come up once these types of scandals are coming to light. First and foremost is that the interest of students already enrolled in various courses rolled out by these dubious universities should be protected. These students should be allowed to continue the same course under some recognized universities immediately so that their academic career is not jeopardized. In the second place, there must be retrospective analysis how this happened and who was involved in this process. It may be a case of sheer incompetence of people involved in this process from top to bottom. Even the public has the right to question the intention and competence of Union Human Resource Development ministry who monitor the system. However, majority of public will suspect involvement of a group of educational mafia in this incidence. Big sum of money must have changed hands at various stages and may be at regular span of time. It brings public accountability for all those who are involved in this type of scam. There should be enquiry in all those cases how these institutes were found eligible to obtain the status of deemed university! Was there any norms or it was simply in exchange of money for few individuals? All cases should be investigated thoroughly to bring out facts in front of the public.

Interesting part is this system failure has been going on for last almost ten years, which involved previous tenure of the same government and also tenure of different political party. Another question comes to the forefront is why Indian public did not raise voice when they were directly involved in this process of education? Why did they tolerate the system of unholy nexus for so long waiting for government to take action? It is difficult to answer. However, it might be due to public acceptance of corruption to facilitate their personal interest. When the ward cannot perform well in qualifying examination, it is easy to get enrolment in these types of deemed universities by paying some hefty amount. Where these “hefty amount” for “donation” comes from? Parents may have earned this money by working hard but are now forced to shell out for their ward's education. Or else, it is a part of unaccounted money coming from another mafia. Money is just changing hands from other mafia to educational mafia.

There is an element of corporate bullying in Indian society. In the era of globalization, it sounds unusual that corporate bully on those people from whom they earn their bread. However, it is true. In early nineties, plastic blood collection bags were not freely available in India. Glass bottles were banned and foreign blood bags were prohibitatively expensive. And one cannot run a blood bank without having blood bags. Due to limited supply, Indian blood bag companies dictated terms. I still remember that my previous government institute had to pay full amount in advance, and then only bags were supplied, which was against purchase norms of the government. Similar situation is still observed when there is monopoly in business. In transfusion medicine, there are technologies like nucleic acid test (NAT), viral inactivation, and few techniques in immunohematolgy are patented. One or two companies are marketing products globally in these areas. Customers are pushed to the wall many a times due to existing monopoly. Prices are exorbitantly high and for a transfusion medicine expert, it is difficult to justify cost benefit ratio after spending huge sum of (public) money.

How can a corporate interfere in the functioning of medical organization? In a recently held national conference, it was quite evident how few corporate houses are throwing weight behind few people who opted for office bearer posts. We heard this type of corporate interference in western medical societies, but it was an eye opener how the other side of the table can turn table on us. That is why I always insist that corporate members should not have voting right in academic societies; otherwise, there will be conflict of interest.

This discussion will not be complete if we do not discuss about decision enforcing people in transfusion medicine in India. In discharging our duties as blood bank officers, managers, or as blood donor motivators, we feel frustrated about influence of some persons as an individual or as a group. A small group of persons is always nearer and dearer to decision/ policy makers, regulatory/assessment agencies, medical education implementing agencies and other medical associations. Persons inherit this status by merit or by credit or by positions held in their place of work. It shows that this organization (*per se* medical community) has vested special responsibility because of faith in this person. It is the responsibility of the person to discharge duties with best of abilities and knowledge in an unbiased manner. If we link our present decisions with past personal relations and future gains, then we will fail in discharging duties. We will deceive that organization (society at large) that has imposed faith on us by virtue of position held in parent organization. It is true when we are holding positions in health ministry or members of associated organizations, regulatory bodies, or professional bodies. Let us all try to develop a clean, vibrant, and technically sound transfusion medicine specialty in India to supply safe blood to the remotest part of the country in sufficient amount. Or else, we may land up in a situation like the fate of 53.7% deemed universities in India.

